# Man vs. machine: comparison of pharmacogenetic expert counselling with a clinical medication support system in a study with 200 genotyped patients

**DOI:** 10.1007/s00228-021-03254-2

**Published:** 2021-12-27

**Authors:** Sally H. Preissner, Paolo Marchetti, Maurizio Simmaco, Björn O. Gohlke, Andreas Eckert, Saskia Preissner, Robert Preissner

**Affiliations:** 1 Department Oral and Maxillofacial Surgery, Charité – Universitätsmedizin Berlin, Corporate Member of Freie Universität Berlin, Humboldt-Universität Zu Berlin, and Berlin Institute of Health, Augustenburger Platz 1, 13353 Berlin, Germany; 2 Department of Neurosciences, Mental Health and Sensory Organs, Faculty of Medicine and Psychology, Sapienza University and Laboratory of Clinical Biochemistry Sant’Andrea Hospital, Rome, Italy; 3 Medical Oncology Unit, Sant’Andrea Hospital, Rome, Italy; 4 Science-IT and Institute of Physiology, Charité – Universitätsmedizin Berlin, corporate member of Freie Universität Berlin, Humboldt-Universität Zu Berlin, and Berlin Institute of Health, Philippstrasse 12, 10115 Berlin, Germany

**Keywords:** Precision medicine, Drug-drug interactions, Polymorphisms, Medication score, Polypharmacy

## Abstract

**Background:**

Medication problems such as strong side effects or inefficacy occur frequently. At our university hospital, a consultation group of specialists takes care of patients suffering from medication problems. Nevertheless, the counselling of poly-treated patients is complex, as it requires the consideration of a large network of interactions between drugs and their targets, their metabolizing enzymes, and their transporters, etc.

**Purpose:**

This study aims to check whether a score-based decision-support system (1) reduces the time and effort and (2) suggests solutions at the same quality level.

**Patients and methods:**

A total of 200 multimorbid, poly-treated patients with medication problems were included. All patients were considered twice: manually, as clinically established, and using the Drug-PIN decision-support system. Besides diagnoses, lab data (kidney, liver), phenotype (age, gender, BMI, habits), and genotype (genetic variants with actionable clinical evidence I or IIa) were considered, to eliminate potentially inappropriate medications and to select individually favourable drugs from existing medication classes. The algorithm is connected to automatically updated knowledge resources to provide reproducible up-to-date decision support.

**Results:**

The average turnaround time for manual poly-therapy counselling per patient ranges from 3 to 6 working hours, while it can be reduced to ten minutes using Drug-PIN. At the same time, the results of the novel computerized approach coincide with the manual approach at a level of > 90%. The holistic medication score can be used to find favourable drugs within a class of drugs and also to judge the severity of medication problems, to identify critical cases early and automatically.

**Conclusion:**

With the computerized version of this approach, it became possible to score all combinations of all alternative drugs from each class of drugs administered (“personalized medication landscape “) and to identify critical patients even before problems are reported (“medication alert”). Careful comparison of manual and score-based results shows that the incomplete manual consideration of genetic specialties and pharmacokinetic conflicts is responsible for most of the (minor) deviations between the two approaches. The meaning of the reduction of working time for experts by about 2 orders of magnitude should not be underestimated, as it enables practical application of personalized medicine in clinical routine.

## Introduction

Improper drug prescription has been recognized as the main societal challenge by The World Health Organization (WHO), and the Third WHO Global Patient Safety Challenge was launched in 2017 [1]. Therapeutic drug therapy is facing various intricacies, which can be summarized in different dimensions of a multi-factorial network [2]. The biological variability, which is drawn by receptors and polymorphic enzymes, forms one of the three dimensions included in the multi-factorial network [3–5]. The second dimension deals with drug-drug interactions (DDIs), which may lead to counterproductive consequences or an decrease in the beneficial effects of the given drugs [6]. The phenotype of the patient, which is effected by age, sex, pattern of comorbidities, habits (e.g., nicotine or alcohol abuse), and physiological features, like liver and kidney function constitutes the third dimension [2]. The consideration of this “personal interaction network” (Drug-PIN) is required to optimize the drug therapy.

Mentionable effort has been made to avoid potentially inappropriate medication [7–9], one of the main issues of drug-cocktail optimization. In particular, age-related problems are considered in the Beers lists and PRISCUS lists [10, 11]. Certain clinically relevant DDIs are listed in drug labels and guidelines. Pharmacogenetically, relevant variances are validated by an international commission (The Clinical Pharmacogenetics Implementation Consortium (CPIC) [12]. Although there are several activities involved in the collection of pharmacogenetic information [13], there is no computational approach integrating all of these aspects into a Clinical Decision Support System (CDSS) for the optimization of medication. Here, we analyse the reproducibility of structured manual drug-cocktail optimizations from a team of experts with CDSS-based results that automatically evaluate all patients’ parameters affecting drug response and to quickly transform such a complex bulk of data into a readable and immediately usable information for appropriate drug selection.

## Material and methods

### Study population

The study included 200 outpatients referred to the Sant’Andrea Hospital of Rome (Table 1); inclusion criteria were a poly-therapy (minimum of four drugs), insufficient response to at least three therapies, and/or adverse drug reactions (ADRs). Informed consent to the processing of personal data was obligatory. Exclusion criteria were minor (≤ 18 years) or advanced age (≥ 75 years), incongruent medications at baseline or during the study period, substantial changes in drug therapy during the study, substance use disorders (except nicotine), neurological (epilepsy, major neurocognitive disorder, Parkinson’s disease), or severe acute organic illnesses (major cardiovascular disorders and hypertension, diabetes, malignancy, renal failure). Informed written consent was signed by each patient before enrolment. The study was approved by the Ethics Committee of the University of Rome and registered under Prot. 987/2014.

**Table 1:** Main characteristics of the cohort

Study population	Percentage (value) (average)	Standard deviation
Age (years)	**56.94**	** + / − 12**
Gender (f/m)	**55/45 (111/89)**	**N/A**
BMI f/m (kg/m^2^)	**24.6/27.4**	** + / − 6**
# of drugs	**8**	** + / − 4**
# of smokers, caffeine and alcohol consumers	**30, 56, 42** **(60, 112, 83)**	**N/A**
GFR (mL/min)	**94**	** + / − 15**
ALT, AST (U/l)	**23, 26**	** + / − 5, + / − 8**
Main diagnoses (ICD-10)	**I11, E11, F32, C50**	**n/a**
# of clinically relevant SNVs	**18**	** + / − 6**

*BMI* body mass index, *GFR* Glomerular filtration rate, *ALT* alanine aminotransferase, *AST* aspartate aminotransferase, *ICD*-10 codes, *I11* hypertensive heart disease, *E11* type 2 diabetes mellitus, *F32* depressive episode, *C50* malignant neoplasm of breast, *SNVs* single-nucleotide variants

### Phenotyping and genotyping of study cohort

All patients were genotyped for 93 polymorphisms in 44 pharmaco-genes, including phase I and phase II drug-metabolizing enzymes, drug transporters, and drug targets [14], using a custom panel designed for targeted DNA re-sequencing using the Ion AmpliSeq™ Library Kit 2.0 chemistry and the next-generation sequencing platform Ion Chef/Ion S5 system (Thermo Fisher Scientific, Waltham, MA, USA) according to the manufacturer’s instructions. DNA samples were obtained from 5 mL of peripheral blood using the QIAsymphony automatic system for nucleic acid extraction (Qiagen, Hilden, Germany).

### Therapy score

**Fig. 1:**
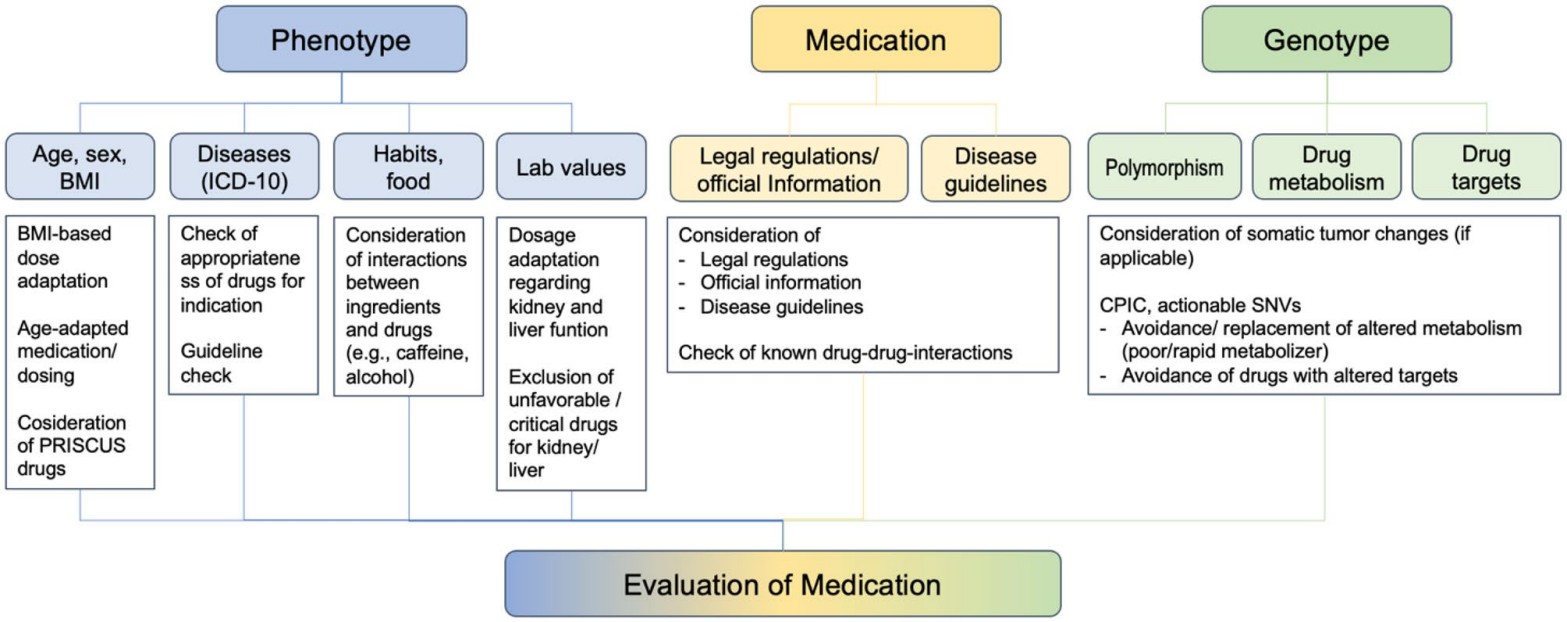
Parameters utilized for the Drug-PIN score

The following parameters are utilized for the development of the Drug-PIN therapy score algorithm (Fig. 1):

- **Phenotype**

- Ethnicity, age, gender, bodyweight
- Lab values (liver/kidney function/albumin/electrolytes);
- Diseases (ICD-10 codes);
- Habits (alcohol, nicotine, caffeine)

- **Genotype**

- Patient’s polymorphisms
- Drug metabolism
- Drug targets
- If applicable (tumour), somatic changes to therapeutic targets
- Clinical relevance of interactions (CPIC)

- **Drug-cocktail**

- Legal regulations (FDA, EMA)
- Official information (drug label, info for HCP)
- Disease-specific guidelines
- Drug metabolism (CYPs (inducer, inhibitor, substrate), phase II enzymes, transporters)
- Food, drinks, herbs

Information from drug labels can lead to a classification either as a problematic drug-drug or drug-gene interaction. Drug-drug interactions are graded in three severity classes (monitor, avoid, contraindicated), which are penalized accordingly (5, 25, 45 points). Drug-gene interactions are treated with more flexibility, depending on the precise genotype, and can range in score penalty from 10, for somewhat problematic interactions, to a maximum of 100 (primarily the case where a polymorphism completely disables a relevant metabolizer with possible fatal toxicity results), as assessed by medical professionals.

In the case of known prodrugs or active metabolites, a warning is displayed to consider those in the assessment. Due to the diverse nature, especially of metabolite products, there is no fixed score for these cases.

In addition to those fixed-term scores, various algorithmic penalties and weight factors contribute to the metabolic component of the score based on expected genetic activity and the nature of known interactions (inhibitors, inducers, substrates).

Phenotype data like BMI influences the expected metabolic activity, while certain habitually consumed products are treated like possible co-medications (ethanol, tobacco ingredients, caffeine).

Additionally, age-based and kidney function-based penalties are applied based on the PRISCUS [15] and Beers lists [16]: 6 levels of kidney activity based on GFR/stages of chronical kidney disease (stages 1, 2, 3a, 3b, 4, 5—with the penalty depending on the stage of disease, going from 0 for healthy to 25 for stage 5)). Liver activity is assessed on the given ALT and AST values in 5 grades, as well as warnings about known conditions like ischemic hepatitis or alcoholic fatty liver disease. Age-related penalties amount to 15 per found interaction. Both PRISCUS and kidney penalties are a little lower, due to various factors at play and a larger component of individuality and doctor’s assessment, so the doctor’s warnings are the primary component here.

The Drug-PIN score is based on penalties for unfavourable interactions and contra-indications. To avoid legal problems with potentially inappropriate medication, rather strong penalties are used for drugs or drug combinations where contra-indications are known. Three generalized interaction risk levels are reported: contra-indicated (indicating that this combination is strongly advised against and should not be used if possible (Drug-Pin score 60–200)), severe (which means modification of the therapy is recommended (Drug-Pin score 20–60)), and moderate (which means monitoring is advisable (Drug-Pin score 0–20)). This slightly coarser system was employed because not all data has the same fidelity, and also to be on the side of caution. In the case of pro-drugs, which can be critical if genetic polymorphisms prevent their activation (e.g., clopidogrel), the software includes a separate warning notification and a penalty for these and other especially noteworthy drug-gene combinations. Further physiology-based terms are summarized under a separate term (age, kidney, liver, protein binding).

Since drug-drug interactions occurring by shared metabolic pathways cannot be correctly evaluated by pairwise considerations for medication composed of more than two drugs, the complete matrix of interactions (Substrate, Inhibitor, Inducer) of all drugs administered with all drug enzymes and transporters has been considered. Drug-PIN provides the opportunity to scan and generate each patient’s standardized national medication plan as a QR code, which is an established procedure in Germany and Switzerland for all patients taking three or more drugs [17] and speeds up the data inclusion significantly.

## Results

### Medication analysis

**Fig. 2:**
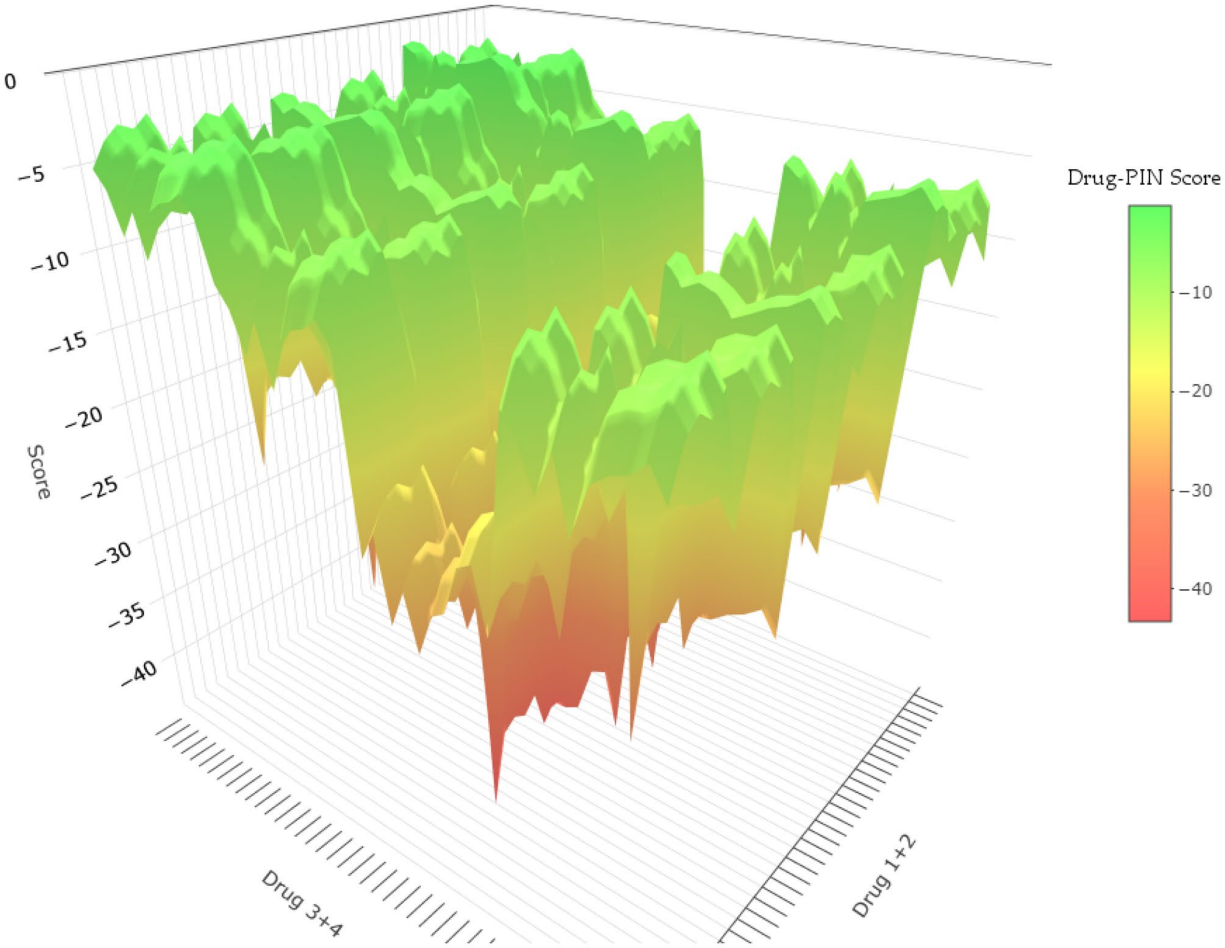
Inverted Drug-PIN score landscape of four common drugs (Citalopram, Atenolol, Pantoprazole, Metamizole). Each data point represents one possible drug combination and its evaluation by the score. In total, 1440 four-drug combinations were assessed by the respective score and constituted the surface. The colour of the surface depends on the score and indicates the severity of problems (traffic-light concept)

The aim of any drug combination is to accomplish an improvement in therapy results, yet the optimization of drug cocktails is a complex problem, even for a comparatively small number of drugs as visualized in Fig. 2. The “score landscape” is rather rugged, and finding the global minimum by one or two drug replacements is unlikely.

Regarding poly-treated patients, the occurrence of severe DDIs ranged from 31.8% in patients taking 6 drugs to more than 60% in patients taking 10 or more drugs (Table 2). Notably, in the same patients’ groups is the percentage for prescribed drugs doubles, which were contra-indicated according to the drug labels and the FDA/EMA regulations (Table 2). That is, if the overall frequency of contra-indicated drugs is about 5%, ranging from 8% in patients taking 6 drugs to 18% in patients taking drugs, there were peaks of about 22% and 33%.

**Table 2:** Frequency of possibly inappropriate medications, split by drug cocktail size and severity of DDIs. Values are given as a percentage

**# of drugs in cocktail**	**Frequency**	**Moderate DDIs**	**Severe DDIs**	**Contra-indicated DDIs**	**Age-related Issues**
1	11.99	-	-	-	0.49
2	15.23	12.80	2.19	0.94	1.52
3	14.21	44.09	7.18	2.15	2.60
4	13.48	65.17	13.44	3.59	4.42
5	12.73	82.06	21.58	5.52	6.30
6	11.76	91.30	31.80	7.97	8.54
7	9.38	95.53	41.94	9.24	10.80
8	5.98	98.23	51.38	11.23	13.26
9	3.35	98.97	58.94	13.98	15.42
10	1.38	99.51	63.40	18.06	15.90
11	0.41	99.71	68.37	22.02	18.56
12	0.11	100.00	67.67	32.33	17.67
ALL	100	58.22	19.86	4.95	5.70

### Score optimization

**Fig. 3:**
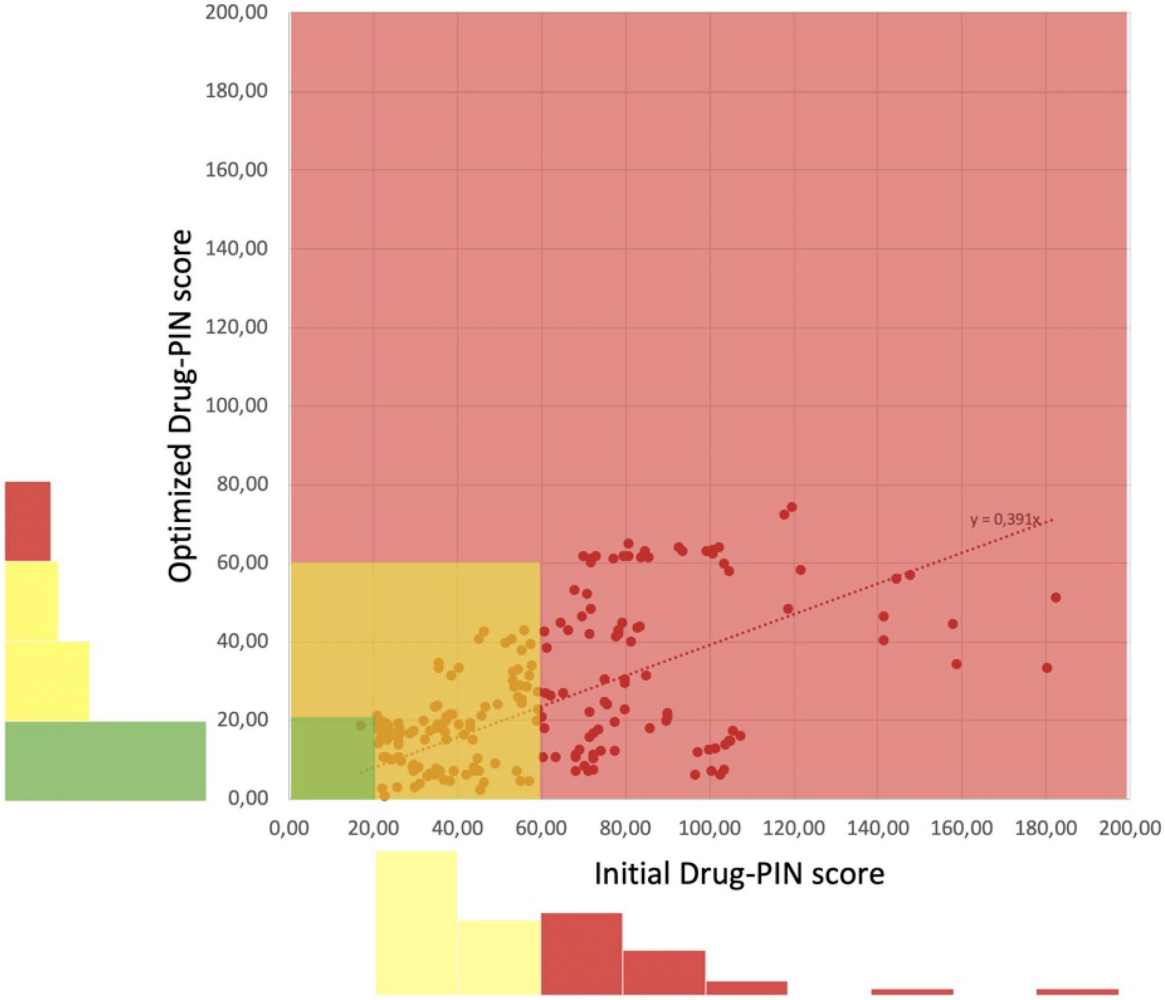
Score distribution of patients with medication problems before and after optimization. Patient scores are reflected by blue dots whereby the position relative to the *x*-axis reflects the score before optimization and the position of the *y*-axis reflects those after optimization. The linear regression shows that the average score is improved by almost 59%. The colour ranges red, yellow, and green, indicate potentially dangerous, moderate, and low DDI levels, respectively. Below and left of the diagram, simple histograms show the frequencies of cocktail scores in potentially dangerous (red bars) and moderate-(yellow), and low (green)-level DDIs

The distribution of score values before and after optimization (Fig. 3) shows that a strong reduction of DDIs can be achieved (score drops by about 60%). While no original patient cocktail shows a low score (<20), about half of all cases fall into this range after optimization. High scores, frequently associated with drugs that cannot be metabolized because of genetic variants in the patient, can often be lowered significantly by the replacement of drugs by other drugs from the same class with a different (intact) metabolic route, the wildtype of the contributing enzymes—not poor or rapid metabolizers. Only about 10% of patients, mostly with a very high number (ten or more) of drugs, remain problematic.

### Comparison of manual vs. automated optimization

**Fig. 4:**
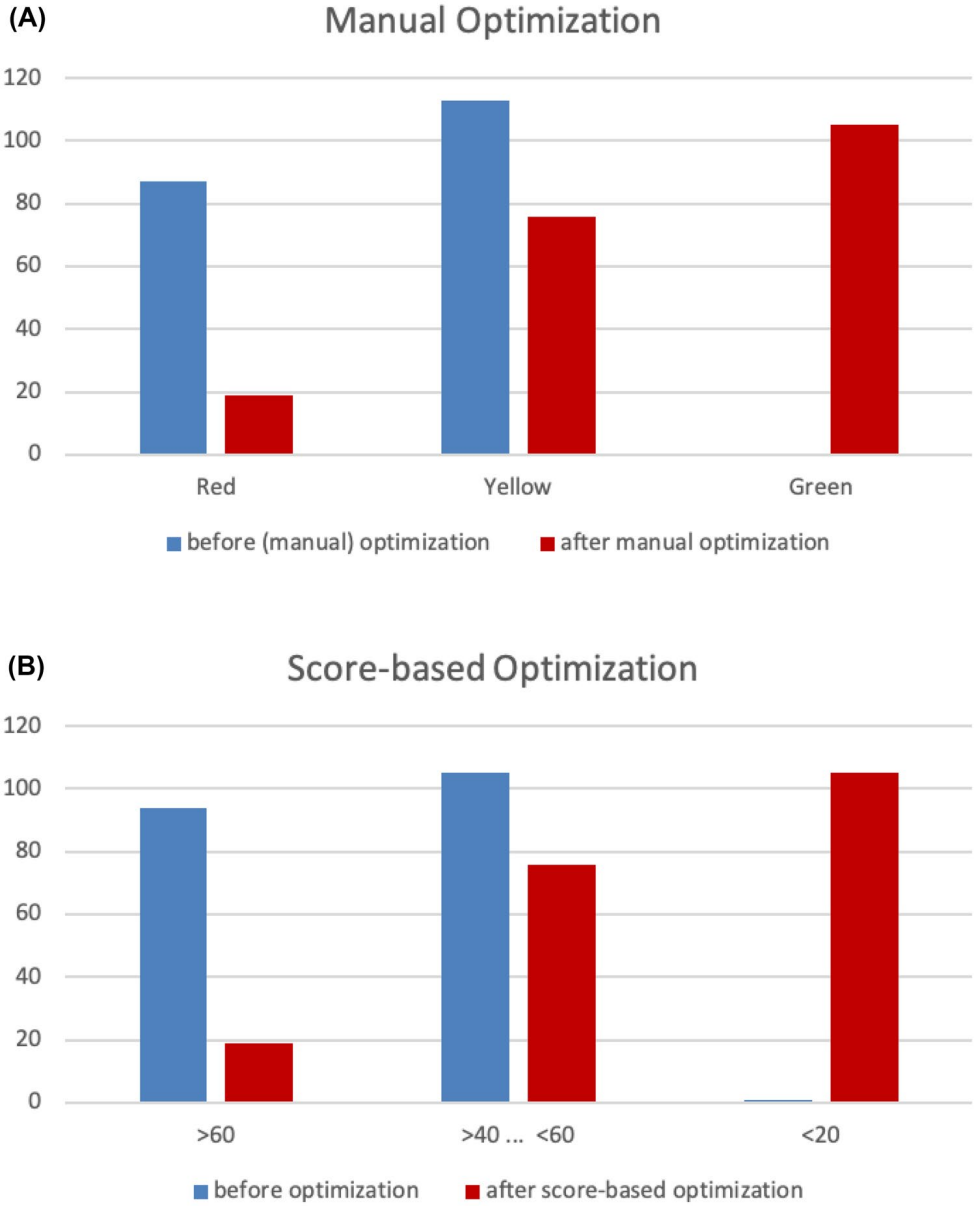
Comparison of manual (**A**) and score-based (**B**) optimization. The two bar graphs compare the manual optimization of drug cocktails with score-based optimization. The blue bars represent the patient’s drug cocktails before optimization, and the red bars symbolize the drug cocktails after optimization, with the Drug-PIN score on the *y*-axis

The automated Drug-PIN score optimization revealed equal results when compared to the manual optimization by specialists (various clinicians—clinical pathologists, pharmacologists, psychiatrists, geneticists) (Fig. 4A, B).

## Discussion

Genomic features affecting the pharmacokinetics and pharmacodynamics of drugs and modifying DDIs have been found to represent a consistent factor increasing the risk associated with drug treatment, being the main medication problem in about 30% of the therapies evaluated in this study. Results from this part of the study are limited by the small number of patients; however, the special features of the small cohort analysed add significance. The size of the examined cohort should be considered as a limiting factor, as well as other confounders, such as the genetic variability of the study population or the variety of diseases of the analysed patients have, as side effects differ within the different drugs prescribed. Non-adherence to the prescribed therapy is also a concern for the present study. However, in the group of patients where baseline therapy was modified according to the Drug-PIN score, a significant improvement of the clinical outcome was obtained. Clinically, this means that the patient’s condition shifts from a moderate disease to a mild disease or from a severe to a moderate disease, which is a promising result in patients who failed to achieve improvement from at least three previous pharmacological therapies.

In general, improper drug use represents one of the most burdening phenomena, causing inefficacy, toxicity, and finally non-adherence to the medical prescription. The huge number of drugs available (about 4000 active ingredients) [18] allows the consideration of deep pheno-/genotyping for the appropriate selection of drugs in poly-therapy [19].

Different research groups have tried to improve medication regimens by developing indices and scores. The Modified Medication Appropriateness Index [20] aims to improve adverse drug reactions, medication regimen complexity, quality of life, and mortality. Pharmacists provide medication reviews following a well-defined scheme, reducing the frequency of drug-related problems [21, 22]. Such reviews are also the basis of an intelligent decision-support system in Australia [23]. The Medication Regimen Complexity Index [24] quantifies the complexity of medication regimens and correlates with hospital readmission and medication adherence while the Medication Regimen Simplification Guide [25] intends to simplify medication by consolidating the number of administration times or using alternative preparations.

All these indices and the CDSS are steps in the right direction but are lacking the integration of patients phenotype (age, size, weight), lab values (e.g., liver and kidney function), habits (e.g., smoking, caffeine, alcohol), and genetic profiles. Using the Drug-PIN score of physiological and genetic factors into a comprehensive matrix of DDIs as suggested here opens the opportunity to improve the compliance as well as the efficacy [17–19].

The Drug-PIN software also embeds criteria from both PRISCUS [26] and Beers [27], suggesting drug alternatives and an age-dependent dose reduction aimed at enabling the application of accepted, but not commonly adopted, recommendations in prescribing pharmacotherapy to elderlies.

The most time-consuming step in the Drug-PIN process is the creation of a patient’s phenotype/genotype including all relevant patient data, like lab values, etc.

The results of this small study show that side effects can be reduced and clinical outcomes can be improved by using Drug-PIN. Although the results of this study seem to be a promising avenue towards prospectively less improper drug prescriptions, a larger, prospective study is certainly needed to confirm this observation and further improve performance.

## Conclusion

The data presented here highlight the importance of a decision-support drug-prescription system for clinical practice. The process of drug prescription must take (less than) a few minutes and is not compatible with the consultation of multiple databases. Drug-PIN software allows the integration of many databases in a unique solution. A substantial reduction in the time needed to evaluate therapy is enabled by Drug-PIN’s replication of the output of the counselling process. The doctor just queries a proposed pharmacotherapy, easily managing alternative drugs arriving at a similar quality level as experienced experts.

## Data Availability

IA Number: PCT/IB2019/052310 “System, Method and Software Program for Managing the Interaction between Drugs”.
